# MicroRNAs 106b and 222 Improve Hyperglycemia in a Mouse Model of Insulin-Deficient Diabetes via Pancreatic β-Cell Proliferation

**DOI:** 10.1016/j.ebiom.2016.12.002

**Published:** 2016-12-07

**Authors:** Sohei Tsukita, Tetsuya Yamada, Kei Takahashi, Yuichiro Munakata, Shinichiro Hosaka, Hironobu Takahashi, Junhong Gao, Yuta Shirai, Shinjiro Kodama, Yoichiro Asai, Takashi Sugisawa, Yumiko Chiba, Keizo Kaneko, Kenji Uno, Shojiro Sawada, Junta Imai, Hideki Katagiri

**Affiliations:** a Department of Metabolism and Diabetes, Tohoku University Graduate School of Medicine, Japan; b Centre for Metabolic Diseases, Tohoku University Graduate School of Medicine, Japan; c Tohoku University Frontier Research Institute for Interdisciplinary Science, Miyagi, Japan.; d Japan Agency for Medical Research and Development, CREST, Japan

**Keywords:** BM, bone marrow, BMT, bone marrow transplantation, CHOP, C/EBP homologous protein, Cip/Kip, CDK interacting protein/kinase inhibitory protein, DAPI, 4′,6-Diamidino-2-phenylindole, GFP, green fluorescent protein, GNZ, a nuclear-localized GFP/LacZ fusion protein, miRNAs, microRNAs, PBS, phosphate-buffered saline, Pri-miR, primary microRNA, STZ, streptozotocin, TM, tamoxifen, TUNEL, Terminal deoxynucleotidyl transferase dUTP nick end labeling, β-cell regeneration, Diabetes, MicroRNAs, Exosomes, Cip/Kip family

## Abstract

Major symptoms of diabetes mellitus manifest, once pancreatic β-cell numbers have become inadequate. Although natural regeneration of β-cells after injury is very limited, bone marrow (BM) transplantation (BMT) promotes their regeneration through undetermined mechanism(s) involving inter-cellular (BM cell-to-β-cell) crosstalk. We found that two microRNAs (miRNAs) contribute to BMT-induced β-cell regeneration. Screening murine miRNAs in serum exosomes after BMT revealed 42 miRNAs to be increased. Two of these miRNAs (miR-106b-5p and miR-222-3p) were shown to be secreted by BM cells and increased in pancreatic islet cells after BMT. Treatment with the corresponding anti-miRNAs inhibited BMT-induced β-cell regeneration. Furthermore, intravenous administration of the corresponding miRNA mimics promoted post-injury β-cell proliferation through Cip/Kip family down-regulation, thereby ameliorating hyperglycemia in mice with insulin-deficient diabetes. Thus, these identified miRNAs may lead to the development of therapeutic strategies for diabetes.

## Highlights

• BMT regenerates β-cells in mice with STZ-induced diabetes and increases miR-106b and miR-222 in serum exosomes and islets.
• Inhibition with anti-miRs against these miRs suppresses BMT-induced β-cell regeneration.
• Injection of miR-106b and miR-222 mimics promotes β-cell proliferation and improves hyperglycemia in STZ-treated mice.

Regeneration of pancreatic β-cells is a promising therapeutic strategy not only for type 1 diabetes but also for certain forms of type 2 diabetes. However, natural regeneration of β-cells hardly ever occurs. Interestingly, bone marrow transplantation (BMT) has been shown to promote β-cell regeneration through an undetermined mechanism(s). In this study, we found that two microRNAs (miR-106b/-222) contribute to BMT-induced β-cell proliferation. Inhibition of miR-106b/-222 using specific anti-miRNAs significantly suppressed BMT-induced β-cell proliferation. Furthermore, intravenously administered miR-106b/222 promoted β-cell proliferation, thereby ameliorating hyperglycemia in mice with insulin-deficient diabetes. Thus, these identified miRNAs may lead to novel therapeutic strategies for diabetes.

## Graphical Abstract

miR-106b-5p and miR-222-3p contribute to post-injury β-cell proliferation through down-regulation of Cip/Kip family members (p21Cip1and p27Kip1).

**Figure:**
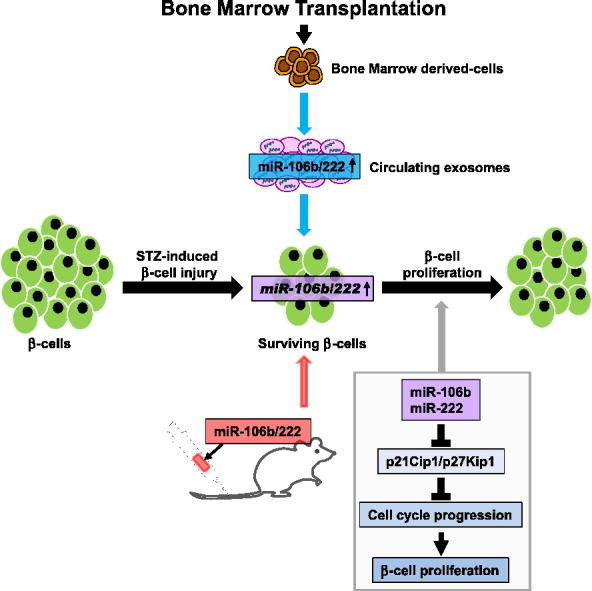

## 1 Introduction

Type 1 diabetes mellitus is characterized by progressive loss of pancreatic β-cells, leading to a life-long dependence on exogenous insulin. In addition, β-cells are reportedly decreased in patients with type 2 diabetes (Butler et al., 2003). In this context, regeneration of pancreatic β-cells is a promising therapeutic strategy for not only type 1 but also some forms of type 2 diabetes. We (Hasegawa et al., 2007) and other groups (Hess et al., 2003, Nakayama et al., 2009) previously reported bone marrow (BM) transplantation (BMT) to promote pancreatic β-cell proliferation after pharmacological β-cell injury, such as that caused by streptozotocin (STZ) treatment. Bone marrow-derived cells were found to have infiltrated sites around regenerating islets (Hess et al., 2003, Hasegawa et al., 2007), suggesting a secretory factor(s) from bone marrow cells to be involved in the underlying mechanism. However, despite intensive research, the specific secretory protein(s)/peptide(s) involved in pancreatic β-cell regeneration after BMT has not as yet been identified.

In addition to secretory proteins, microRNAs (miRNAs) which are transported in exosomes have recently attracted considerable attention for their roles in inter-cellular communication. Exosomes are lipid nano-vesicles, and are secreted by many types of cells and contain a variety of molecules including miRNAs (Turchinovich et al., 2013, Hirsch et al., 2013). miRNAs are a class of endogenous, small, non-coding RNAs that negatively regulate gene expression via translational inhibition or degradation of their target mRNAs (Bartel, 2009). miRNAs have been shown to regulate basic cellular functions including cell proliferation, differentiation, and death (Shenoy and Blelloch, 2014). Therefore, we hypothesized that miRNAs transferred into exosomes mediate the mechanism underlying β-cell regeneration in response to BMT, and comprehensively examined miRNA levels in serum exosomes in BMT-mice. We found that two microRNAs, miR-106b-5p and miR-222-3p, contribute to BMT-induced β-cell regeneration. Furthermore, intravenous administration of the corresponding miRNA mimics promoted post-injury β-cell proliferation, thereby ameliorating hyperglycemia of insulin-deficient diabetes.

## 2 Materials and Methods

### 2.1 Animals

C57BL/6J mice (Clea Japan, Tokyo, Japan), RIP-CreER mice (Stock Number: 008122, The Jackson Laboratory, ME, USA) and Rosa26-GNZ mice (Stock Number: 008516, The Jackson Laboratory) were obtained, housed in an air-conditioned environment, with a 12/12-hour light-dark cycle, and ad lib access to a regular unrestricted diet. Hyperglycemia was induced by intraperitoneal infusion of 50 mg/kg body weight STZ (Sigma, MO, USA) for 5 consecutive days. STZ was dissolved in 0.05 M citrate sodium buffer (pH 4.5) and injected into 6-week-old mice. Tamoxifen (TM) (Sigma) was dissolved at 20 mg/ml in corn oil (Sigma) by sonication at room temperature. TM was injected intraperitoneally at 80 mg/kg body weight daily for 5 consecutive days. This study was approved by the ethics committees of Tohoku University. All animal experiments were conducted in accordance with Tohoku University institutional guidelines.

### 2.2 Measurements

Blood glucose was measured after a 9-hour daytime fast and assayed using Glutest Mint (Sanwa Chemical, Aichi, Japan). Plasma insulin concentrations were determined with an ELISA kit (Morinaga, Kanagawa, Japan). For measurement of the pancreatic insulin content, samples of the pancreas were suspended in ice-cold acid–ethanol (0.18 M HCl in 75% ethanol) and minced with scissors, and left at − 20 °C for 48 h, with sonication every 24 h. The supernatant of each sample was diluted with a solution of 1 mM EDTA/1% bovine serum albumin in phosphate-buffered saline (PBS) and subjected to the ELISA assay (Kondo et al., 2013).

### 2.3 Bone Marrow Transplantation (BMT)

BM cells were collected by dissecting the femurs and tibias, removing excess tissue, cutting the ends of the bones and flushing out the BM with PBS. Then, BM cells were filtrated through a Nylon cell strainer (BD Biosciences, CA, USA). The BM donors were 6-week-old C57BL/6J mice. Recipient mice were lethally irradiated with 10 Gy and reconstituted with a single injection of 4 × 10^6^ donor BM cells through the tail vein (Hasegawa et al., 2007).

### 2.4 Preparation of Synthetic miRNAs/Atelocollagen Complex

miRNA mimics (miR-106b, miR-222 and non-targeting control) (mirVana miRNA mimics, Ambion, TX, USA), anti-miRNAs (anti-miR-106b-5p, anti-miR-222-3p and non-targeting control) (mirVana miRNA inhibitors, Ambion) or fluorescein-labeled miRNA mimics (miR-106b and miR-222) (Cosmo Bio, Tokyo, Japan) were used in the experiments. For preparation of the synthetic miRNAs/atelocollagen complex (AteloGene; Koken, Tokyo, Japan), equal volumes of atelocollagen and miRNA solution were combined and mixed, following the manufacturer's protocol. Each mouse was restrained and slowly administered 200 μl of mixtures, containing 4 nmol of miRNA mimics (2 nmol of miR-106b and 2 nmol of miR-222), 4 nmol of anti-miRNAs (2 nmol of anti-miR-106b-5p and 2 nmol of anti-miR-222-3p) or 4 nmol of the non-targeting control, via the tail-vein using a disposable insulin syringe (TERUMO Myjector 29G × 1/2″, TERUMO, Tokyo, Japan). The injection speed was < 5 μl/s.

### 2.5 Analyses of Pancreases after Intravenous Administration of Fluorescein-Labeled miRNAs with Atelocollagen

Fluorescein-labeled miRNA mimics (2 nmol of miR-106b and 2 nmol of miR-222) mixed with atelocollagen were injected into the tail veins of STZ-treated mice. After 24 and 48 h, pancreases were removed and embedded in OCT compound and frozen. Cryostat pancreatic sections (8 μm thick) were placed on microscope slides, stained with DAPI (SouthernBiotech, AL, USA) and observed using a fluorescence microscope (BZ-9000, Keyence, Osaka, Japan).

### 2.6 Exosome Isolation

Exosomes from mouse serum were isolated using ExoQuick solutions (System Biosciences., CA, USA) following the manufacturer's protocol. Briefly, 1/4 volume of ExoQuick was added to serum and the samples were refrigerated at 4 °C for 30 min. Next, exosomes were precipitated by centrifugation at 1500 ×* g* for 30 min and the supernatant was removed by aspiration. Exosomes from cell culture media were isolated using ExoQuick-TC solutions (System Biosciences) following the manufacturer's protocol. Briefly, cell culture media were centrifuged at 3000 ×* g* for 15 min to remove cells and cell debris. After filtration through 0.22 μm pore size filters (Millex-GV Filter, Merck Millipore, Darmstadt, Germany), 10 ml of cell culture supernatant were mixed with 2 ml of Exoquick-TC and refrigerated overnight. Next, exosomes were precipitated by centrifugation at 1500 ×* g* for 30 min and the supernatant was removed by aspiration.

### 2.7 Isolation and Culture of BM Cells

Six days after BMT, BM cells were harvested from femurs and tibias and cultured in Dulbecco modified Eagle medium containing 10% exosome-depleted fetal bovine serum media (System Biosciences) and penicillin-streptomycin at 37 °C and 5% CO_2_. BM cells were plated in 12 ml of cell culture medium on collagen type-1 coated 10 cm dishes (Iwaki, Tokyo, Japan). After 10 h of cell culture, media were collected and exosomes were isolated as described above.

### 2.8 Primary Islet Cell Culture and Transfection

Pancreatic islets were isolated from 10- to 11-week-old C57BL/6J mice by retrograde injection of collagenase (Sigma) into the pancreatic duct according to the standard procedure, as described previously (Imai et al., 2008, Gotoh et al., 1985). The freshly isolated islets were dissociated into dispersed islet cells by trypsinization and distributed into 96-well plates (40 islets per well) and maintained in RPMI1640 medium containing 10% fetal bovine serum, penicillin-streptomycin and gentamicin at 37 °C and 5% CO_2_ for 2 days. Then, islet cells were co-transfected with 10 pmoles of miR-106b mimics and 10 pmoles of miR-222 mimics (Ambion), or transfected with 20 pmoles of non-targeting control (Ambion) using Lipofectamine RNAiMAX (Invitrogen) according to the manufacturer's instructions. Three days after transfection, the cells were collected and analyzed for Ki-67 mRNA expression.

### 2.9 Immunohistochemistry

The pancreases were excised, fixed overnight in 10% paraformaldehyde and embedded in paraffin. Samples were sectioned at 3 μm and stained with hematoxylin-eosin or incubated with primary antibodies: p21Cip1(ab2961, Abcam, Cambridge, UK), p27Kip1(ab7961, Abcam), p57Kip1(ab75974, Abcam), p53 (NCL-p53-CM5p, Leica Biosystems, Wetzlar, Germany), CHOP (sc-575, Santa Cruz Biotechnology, CA, USA), GFP (sc-8334, Santa Cruz), Ki-67 (#12202, Cell Signaling Technology, MA, USA), insulin (I2018, Sigma) or glucagon (A0565, Dako, CA, USA). The immune complexes were visualized with DAB (Histofine Simple Stain Mouse MAX-PO (R) or Histofine Mouse Stain Kit; Nichirei, Tokyo, Japan). Alexa Fluor 488 goat anti-mouse IgG (Sigma) or Alexa Fluor 546 goat anti-rabbit IgG (Dako) was used as the fluorescent secondary antibody. Dapi-Fluoromount-G™ (Southern Biotech, AL, USA) was used to stain nuclei in the final step. At least 20 islets with > 1000 islet cells were counted per mouse for evaluation of p21, p27, p53, CHOP, GNZ and Ki-67 expression by IHC staining. GNZ protein was stained with anti-GFP antibody. Both positive and negative cells were counted manually using the ImageJ Cell Counter plugin.

### 2.10 Terminal Deoxynucleotidyl Transferase dUTP Nick End Labeling (TUNEL) Assay

The TUNEL assay was performed to detect DNA fragmentation associated with apoptosis using an in situ cell death detection kit (Roche Applied Science, Mannheim, Germany). The results are expressed as the mean number of TUNEL-positive nuclei per islet.

### 2.11 Laser Capture Microdissection (LMD) of Islets

Seven days after BMT, pancreases were removed, embedded in OCT compound and flash-frozen. Samples were stored at − 80 °C until cryostat sectioning. Cryostat pancreatic sections (10 μm thick) were placed on PEN-coated slides (Leica Microsystems, Wetzlar, Germany) and stained with hematoxylin, allowing islets to be recognized. Immediately after staining, LMD was carried out on a Leica AS LMD (Leica Microsystems) (Tsukita et al., 2012).

### 2.12 RNA Extraction

RNA from exosomes was extracted using the SeraMir Exosome RNA Purification Column Kit (System Biosciences) following the manufacturer's protocol. RNA from BM cells was extracted using the miRNeasy Micro Kit (QIAGEN) following the manufacturer's protocol. miRNA was extracted from approximately 3 × 10^5^ femoral BM cells. RNA from laser capture microdissected islets was extracted using the miRNeasy Micro Kit (QIAGEN) following the manufacturer's protocol. RNA from cultured islet cells was extracted using the RNeasy Micro Kit (QIAGEN).

### 2.13 Quantification of Mature miRNAs by RT-PCR

miRNA was converted to cDNA using the QuantiMir RT kit (System Biosciences) following the manufacturer's protocol. Briefly, RNA was tagged with poly(A) tails and reverse transcribed using poly(T) adapter primer. For each miRNA detection, a mature DNA sequence was used as the forward primer and a 3′ universal reverse primer was provided by the QuantiMir RT kit. Mouse U6 snRNA was amplified as an internal control. qPCR using Power SYBR Green Mastermix (Applied Biosystems, Warrington, UK) was performed employing the 7900HT Fast Real Time PCR system (Applied Biosystems). The amplification conditions were as follows: 1 cycle of 50 °C for 2 min and 1 cycle of 95 °C for 10 min, followed by 40 cycles of 95 °C for 15 s and finally 60 °C for 1 min. Specificity was verified by melt curve analysis. Relative quantification of each miRNA was performed using the comparative Ct method.

### 2.14 Quantification of Primary miRNAs and mRNAs by RT-PCR

After DNase treatment using the TURBO DNA-free Kit (Ambion), RNAs extracted from islets were converted to cDNA using the High Capacity RNA-to-cDNA Kit (Applied Biosystems). Expressions of the primary miRNAs were analyzed using TaqMan Pri-miRNA assay (pri-miR-106b Assay ID Mm03306675_pri, pri-miR-222 Assay ID Mm03307187; Applied Biosystems). Ki-67 mRNA expression was analyzed using the TaqMan Gene Expression Assay (Assay ID Mm01278617_m1). GAPDH (Assay ID Mm99999915_g1) and β-actin (Assay ID Mm00607939_s1) were amplified as internal controls. qPCR was performed on the 7900HT Fast Real Time PCR system (Applied Biosystems). The amplification conditions were as follows: 1 cycle of 50 °C for 2 min and 1 cycle of 95 °C for 10 min, followed by 40 cycles of 95 °C for 15 s, and 60 °C for 1 min. Specificity was verified by melt curve analysis. Relative quantification of primary miRNAs and mRNAs was performed using the comparative Ct method.

### 2.15 Screening for miRNA by qPCR Analysis

The miRNA expression profiles were compared between BMT-mice and control mice (without irradiation and infusion of BM cells) using the miRNome microRNA profiler (System Biosciences) following the manufacturer's protocol. The sera of 3 mice from each group were pooled and counted as one sample. Serum exosomes of BMT-mice were collected 6 days after BMT as described above. RNA extraction and cDNA synthesis were performed as described above. Real-time qPCR was performed with cDNA synthesized from miRNAs, 2 × SYBR Green qPCR Mastermix buffer and primers. Specific forward primers for 709 mouse miRNAs and universal reverse primer were provided by the manufacturer. Three endogenous housekeeping RNAs, U6 snRNA, RNU43 and U1, were run on each plate and served as reference controls. qPCR was performed on the 7900HT Fast Real Time PCR system in 384-well qPCR plate. Data analysis was performed using the comparative Ct calculation software provided by the manufacturer.

### 2.16 Statistical Analysis

All data are expressed as means ± SEM. The statistical significance of differences was assessed using the two-tailed unpaired *t-*test or ANOVA followed by multiple comparisons, as appropriate.

## 3 Results

### 3.1 BMT Increases miR-106b-5p and miR-222-3p Levels in Serum Exosomes

**Fig. 1:**
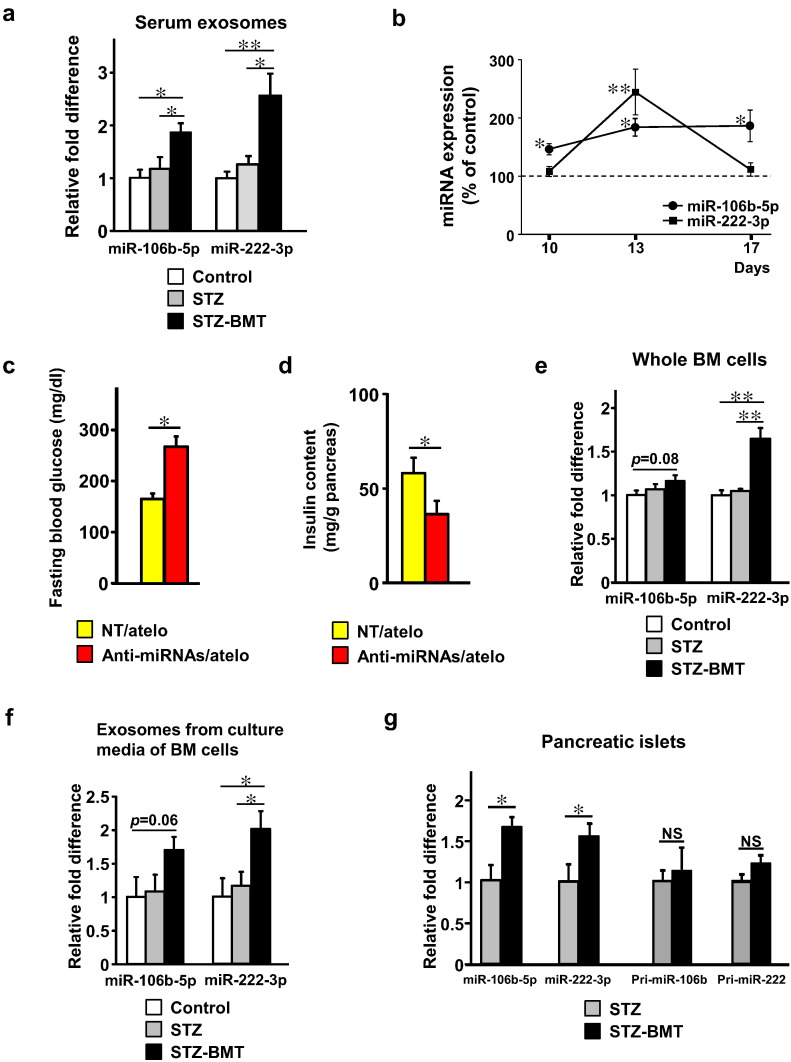
miR-106b-5p and miR-222-3p in serum exosomes are secreted by BM-cells and mediate β-cell regeneration after BMT. (a) The expression levels of miR-106b-5p and miR-222-3p in serum exosomes. Mice received i.p. injections of STZ for 5 consecutive days starting on day 1. BMT was performed on day 7. Serum exosomes were isolated on day 13. Control n = 4, STZ n = 4, STZ-BMT n = 5. Data are presented as means ± SEM; and * *p* < 0.05, ** *p* < 0.01. (b) The expression levels of miR-106b-5p and miR-222-3p in serum exosomes. Mice received i.p. injections of STZ for 5 consecutive days starting on day 1. BMT was performed on day 7. Serum exosomes were isolated on days 10, 13 and 17. Results are expressed as a percentage of the control value. Control n = 5 (day 10), n = 4 (day 13), n = 5 (day 17); STZ-BMT n = 6 (day 10), n = 5 (day 13), n = 5 (day 17). Data are presented as means ± SEM; and * *p* < 0.05 vs. control, ** *p* < 0.01 vs. control. (c) Fasting blood glucose on day 15. STZ-BMT-mice were treated with non-targeting control mixed with atelocollagen (NT/atelo) or anti-miR-106b-5p and anti-miR-222-3p mixed with atelocollagen (Anti-miRNAs/atelo) on days 8, 11 and 14. NT/atelo n = 6, Anti-miRNAs/atelo n = 7. (d) Pancreatic insulin content on day 50. NT/atelo n = 6, Anti-miRNAs/atelo n = 7. (e) The expression levels of miR-106b-5p and miR-222-3p in total BM cells. BM cells were isolated on day 14. n = 6 per group. (f) The expression levels of miR-106b-5p and miR-222-3p in exosomes isolated from the culture media of BM cells. n = 8 per group. (g) The expression levels of miR-106b-5p, miR-222-3p, pri-miR-106b and pri-miR-222 in pancreatic islets. n = 5 per group. Data are presented as means ± SEM; and * *p* < 0.05, ** *p* < 0.01, NS, not significant.

As we reported previously, lethal irradiation and subsequent infusion of BM cells following STZ administration for 5 days (STZ-BMT) resulted in significant improvements of blood glucose levels as compared with STZ treatment alone (STZ) (Supplementary Fig. 1a) with higher pancreatic insulin contents (Supplementary Fig. 1b). Using BMT-mice, we screened 709 murine miRNAs in serum exosomes using miRNA qPCR analysis 6 days after BMT. Expression levels of the 709 miRNAs are shown in Supplementary Table 1. Supplementary Table 2 additionally lists miRNAs with absolute fold changes > 3. We found 42 and 19 miRNAs to be up-regulated and down-regulated, respectively (Supplementary Table 2). Among them, miR-106b and 222 reportedly suppress expressions of Cip/Kip family members (p21Cip1, p27Kip1 and p57Kip2) (Kim et al., 2009, Le Sage et al., 2007, Ivanovska et al., 2008), which negatively regulate β-cell proliferation by inhibiting cell cycle progression (Avrahami et al., 2014, Blandino-Rosano et al., 2012, Georgia and Bhushan, 2006, Zhong et al., 2007). Furthermore, proliferation of pancreatic β-cells was significantly enhanced in p27Kip1 knockout mice as compared to WT mice after STZ treatment (Georgia and Bhushan, 2006). In addition, upregulations of miR-106b and 222 were especially high, with increases of 7.85-fold and 4.45-fold, respectively. Taking these observations together, we hypothesized that these miRNAs contribute to β cell proliferation after BMT. We quantitatively confirmed that STZ-BMT-mice exhibited significantly higher levels of exosomal miR-106b-5p and miR-222-3p in their sera than controls, by 2- and 2.5-fold, respectively, while STZ-mice did not, on day 13 after the first STZ administration (6 days after BMT) (Fig. 1a). Additionally, analysis of these miRNA expressions on 3 separate days after BMT revealed that miR-106b-5p levels remained consistently elevated after BMT. In contrast, miR-222-3p was transiently increased and then decreased, reaching levels similar to those of control mice 10 days after BMT (Fig. 1b).

### 3.2 Inhibition of miR-106b-5p and miR-222-3p Suppresses BMT-Induced β-Cell Regeneration

Then, we examined whether these miRNA elevations contribute to the post-injury β-cell regeneration induced by BMT. In an attempt to block endogenous miR-106b-5p and miR-222-3p functions, we intravenously administered anti-miRNAs specific for miR-106b-5p and miR-222-3p after BMT. In this experiment, we mixed anti-miRNAs with atelocollagen to achieve in vivo stabilization and delivery (Nakasa et al., 2011, Sun et al., 2012). Administration of anti-miRNAs to STZ-BMT mice significantly raised blood glucose levels as compared with non-targeting control administration (Fig. 1c). In addition, pancreatic insulin contents of anti-miRNA-treated STZ-BMT-mice were significantly lower than those of non-targeting control-treated STZ-BMT-mice on day 50 (Fig. 1d). These results suggest that miR-106b-5p and miR-222-3p, with increased serum levels, play important roles in BMT-induced β-cell regeneration.

### 3.3 miR-106b-5p and miR-222-3p are Secreted by BM-cells, and are Increased in Pancreatic Islets after BMT

Next, we examined whether BM cells do, in fact, produce these miRNAs. Expressions of miR-106b-5p and miR-222-3p in total BM-cells tended to be and were significantly increased, respectively, in STZ-BMT-mice as compared with STZ-mice (Fig. 1e). Consistent with their expressions in BM cells, quantification of the levels of these two miRNAs in cultured media of BM cells revealed that miR-106b-5p tended to be increased and that miR-222-3p was significantly increased in exosomes from culture media of BM cells obtained from STZ-BMT-mice as compared with STZ-mice (Fig. 1f). Thus, BM cells produce these miRNAs and secrete them via exosomes. BMT is likely to trigger increased production/secretion of these miRNAs by BM cells.

The next question was whether these miRNAs were actually transferred into β-cells after acute injury. We isolated pancreatic islets by laser-microdissection from STZ-mice and STZ-BMT-mice (Supplementary Fig. 1c), followed by analyzing the expressions of these mature miRNAs as well as primary transcripts of both miRNAs in islets. BMT significantly increased these mature miRNAs in islets (Fig. 1g). In contrast, islet expressions of primary transcripts of these miRNAs, pri-miR-106b and pri-miR-222, were not affected by BMT. These findings suggest that BMT increases miR-106b-5p and miR-222-3p in islets due to transfer from the external environment of islet cells, such as via circulating exosomes and/or exosomes secreted from BM cells infiltrating the areas around regenerating islets.

### 3.4 Exogenous Administration of miR-106b and miR-222 Promotes Post-Injury β-Cell Regeneration, thereby Ameliorating Diabetes

**Fig. 2:**
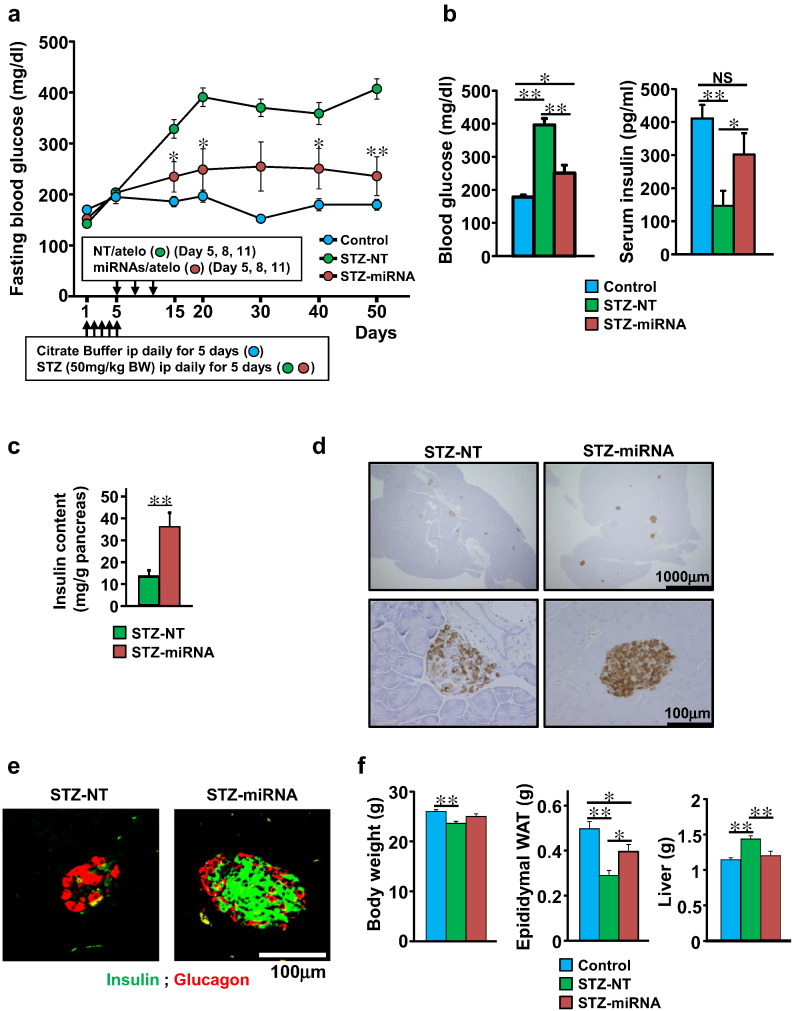
Intravenous administration of miR-106b and miR-222 improves hyperglycemia in STZ-treated diabetic mice. (a–f) Mice received i.p. injections of STZ for 5 consecutive days starting on day 1. Non-targeting control mixed with atelocollagen (NT/atelo) or miR-106b and miR-222 mimics mixed with atelocollagen (miRNA/atelo) were intravenously injected into STZ-mice on days 5, 8 and 11. Mice were sacrificed for analysis on day 50. Scale bar, 100 μm. Data are presented as means ± SEM; and * *p* < 0.05, ** *p* < 0.01, NS, not significant. (a) Fasting blood glucose of STZ-mice treated with non-targeting control mixed with atelocollagen (STZ-NT) or miR-106b and miR-222 mimics mixed with atelocollagen (STZ-miRNA). Control, citrate buffer-treated mice given neither STZ nor miRNA mimics. Control n = 6, STZ-NT n = 8, STZ-miRNA n = 7. *p* value for STZ-NT vs. STZ-miRNA. (b) Fasting blood glucose and serum insulin levels on day 45. Control n = 6, STZ-NT n = 8, STZ-miRNA n = 7. (c) Pancreatic insulin content on day 50. STZ-NT n = 8, STZ-miRNA n = 7. (d) Representative images of pancreatic sections stained for insulin on day 50. (e) Representative fluorescence images of pancreatic islets stained for insulin (green) and glucagon (red) on day 50. (f) Body weight, epididymal white adipose tissue (WAT) weight and liver weight on day 50. Control n = 6, STZ-NT n = 8, STZ-miRNA n = 7.

Therefore, we postulated that administering these miRNAs would be a promising strategy for promoting post-injury β-cell regeneration. To address this issue, miR-106b and miR-222 mimics mixed with atelocollagen (miRNAs/atelo) were intravenously injected into STZ-mice on days 5, 8 and 11 after initial STZ administration. Interestingly, the fluorescein-labeled miRNAs/atelo were selectively delivered to islet cells in the pancreases of STZ-mice (Supplementary Fig. 2a–c). miRNAs/atelo treatment (STZ-miRNA-mice) significantly lowered fasting blood glucose on day 15 after initial STZ administration and lower glucose levels persisted through at least day 50 (Fig. 2a). Fasting serum insulin levels on day 45 and pancreatic insulin contents on day 50 were significantly increased in STZ-miRNA - as compared to STZ-NT-mice (Fig. 2b and c). Anti-insulin staining of pancreatic specimens on day 50 revealed that insulin-positive cells were markedly increased (Fig. 2d). In addition, double immunostaining against insulin and glucagon demonstrated that insulin-positive cells were abundant in the core, and glucagon-positive cells were found in the mantle, of islets from STZ-miRNA-mice, while far fewer insulin-positive cells were detected in the islets of STZ-NT-mice (Fig. 2e). Although STZ-NT-mice showed reductions in body weight and adiposity as compared with controls on day 50, these abnormalities were attenuated in STZ-miRNA-mice (Fig. 2f), indicating recovery from insulin deficiency. Thus, systemic administration of miR-106b and miR-222 mimics promotes post-injury β-cell regeneration, thereby ameliorating insulin-deficient diabetes.

In addition to the pancreas, we examined the distribution of miRNAs/atelo in the liver, kidneys and heart 24 h after injection. As shown in Supplementary Fig. 2d–f, strong fluorescent signals surrounding the portal veins were observed, indicating accumulation of miRNAs/atelo in the liver, while fluorescence was scarce in the kidneys and heart. On day 50, the livers of STZ-NT-mice weighed more than those of control mice. In contrast, liver weights of STZ-miRNA mice were equivalent to those of control mice (Fig. 2f). Hepatomegaly is reportedly associated with STZ-induced diabetes, and is reversed by insulin treatment (Kume et al., 1994, Herrman et al., 1999). Taken together, these results suggest that miRNAs/atelo administration prevents insulin deficiency effects on hepatocytes, rather than inducing hepatocyte proliferation.

### 3.5 Exogenous Administration of miR-106b and miR-222 down-Regulates the Expression of Cip/Kip Family Members in Islets

**Fig. 3:**
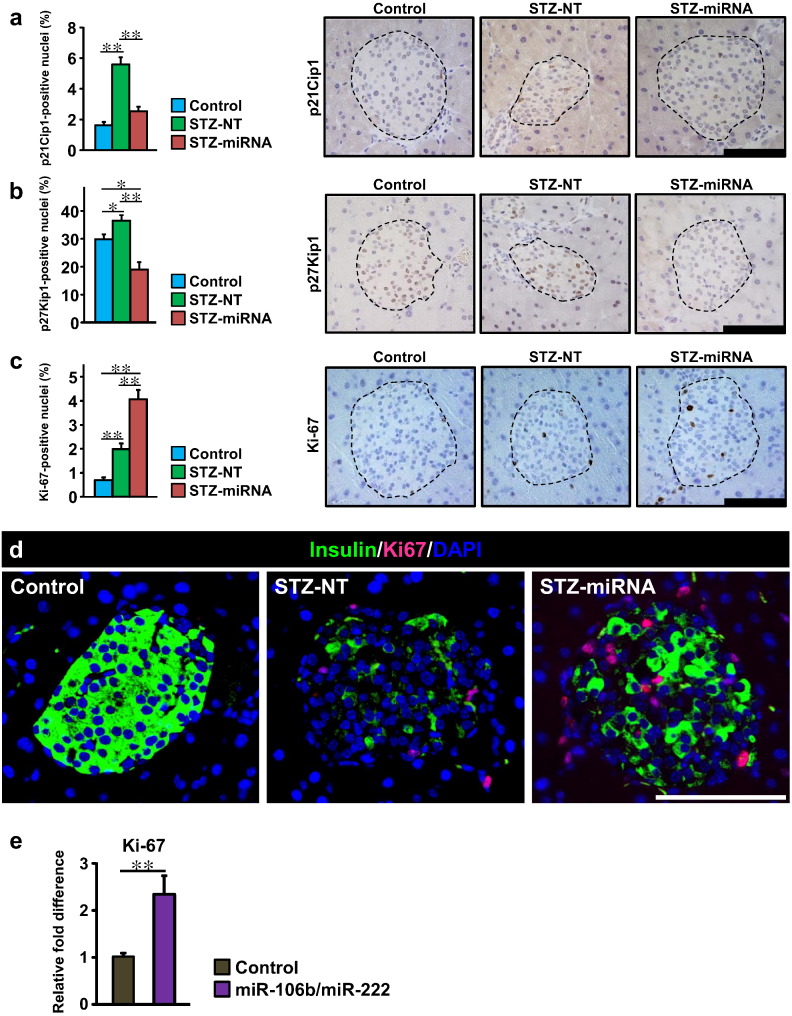
Recovery from acute β-cell injury is due to increased β-cell proliferation through downregulation of members of the Cip/Kip family. (a–d) Mice received i.p. injections of STZ for 5 consecutive days starting on day 1. Non-targeting control mixed with atelocollagen (STZ-NT) or miR-106b and miR-222 mimics mixed with atelocollagen (STZ-miRNA) were intravenously injected into STZ-mice on days 5 and 8. Mice were sacrificed for histological study on day 9. Scale bar, 100 μm. Data are presented as means ± SEM; and * *p* < 0.05, ** *p* < 0.01. (a) Percentage of p21Cip1-positive islet nuclei (left) and representative images of pancreatic islets stained for p21Cip1 (right) on day 9; islets are surrounded by a dashed line. n = 4 per group. (b) Percentage of p27Kip1-positive islet nuclei (left) and representative images of pancreatic islets stained for p27Kip1 (right) on day 9. n = 4 per group. (c) Percentage of Ki-67-positive islet nuclei (left) and representative images of pancreatic islets stained for Ki-67 (right) on day 9. n = 4 per group. (d) Representative images of pancreatic islets stained for insulin (green), Ki-67 (red) and DAPI (blue). (e) The expression levels of Ki-67 in primary islet cells isolated from C57BL/6J mice. Primary islet cells were co-transfected with miR-106b and miR-222 mimics, or transfected with non-targeting control, and then cultured for 3 days. They were then collected and mRNA levels of Ki-67 were determined by RT-PCR using β-actin as an internal control. n = 6 per group. Data are presented as means ± SEM; and ** *p* < 0.01.

To clarify the mechanism(s) whereby administering synthetic mimics of miR-106b and miR-222 causes β-cell regeneration, we first examined pancreatic expressions of Cip/Kip family members (p21Cip1, p27Kip1 and p57Kip2), which are reportedly down-regulated by these miRNAs (Bueno and Malumbres, 2011). As previously reported (Heit et al., 2006), expression of p57Kip2 was not detectable in islet cells of Control, STZ-NT or STZ-miRNA mice. On the other hand, on day 9 after the first STZ administration, p21Cip1- and p27Kip1-positive cells were significantly increased in islets (Fig. 3a and b), suggesting up-regulations of these Cip/Kip family members to suppress post-injury β-cell regeneration in STZ-NT mice. Meanwhile, intravenous administration of the synthetic miRNA mimics significantly decreased p21Cip1- and p27Kip1-positive cells in pancreatic islets (Fig. 3a and b). Intriguingly, Ki-67 immunostaining revealed marked enhancement of islet cell proliferation in STZ-miRNA-mice (Fig. 3c). In contrast, there were no significant differences in ratios of TUNEL-positive cells between islets of STZ-NT-mice and STZ-miRNA-mice (Supplementary Fig. 3a). In addition, no significant differences in the frequencies of p53- and C/EBP homologous protein (CHOP)-positive islet cells were observed between STZ-NT-mice and STZ-miRNA-mice (Supplementary Fig. 3b and c). Collectively, these results indicate that administering synthetic miRNA mimics for miR-106b and miR-222 increased pancreatic β-cells due mainly to promoting proliferation, rather than suppression of apoptosis. Suppressing expressions of Cip/Kip family members is likely to play an important role in the underlying mechanism.

We further performed triple staining with DAPI and the antibodies against Ki-67 and insulin on day 9. At this time-point, STZ treatment (STZ-NT mice) dramatically decreased insulin expression rather than decreasing the cell numbers in pancreatic islets (Fig. 3d). Despite this condition, in STZ-miRNA mice, insulin-expressing β-cells were relatively preserved and a major portion of the islet cell population which was positive for Ki-67 in the nuclei was also positive for insulin in the cytoplasm (Fig. 3d). Considering that STZ treatment reportedly promotes degranulation of insulin vesicles in surviving β-cells (Eleazu et al., 2013, Masson et al., 2009), insulin-expressing and insulin vesicle-degranulated β-cells are likely to undergo proliferation in STZ-miRNA mice. In addition to these in vivo experiments, we further examined the effects of miR-106b and miR-222 mimics on cellular proliferation using islet cells isolated from mice. As shown in Fig. 3e, co-transfection of isolated islet cells with miR-106b and miR-222 mimics significantly increased the expression levels of Ki-67. Thus, these miRNA mimics have the ability to directly promote β-cell proliferation. Taken together, these findings suggest that the miRNA mimics, delivered to pancreatic β-cells injured by STZ, down-regulated p21Cip1 and p27Kip1, thereby promoting β-cell proliferation.

### 3.6 Proliferation of Residual β-Cells Is the Predominant Mechanism of β-Cell Regeneration Induced by miRNA Administrations

**Fig. 4:**
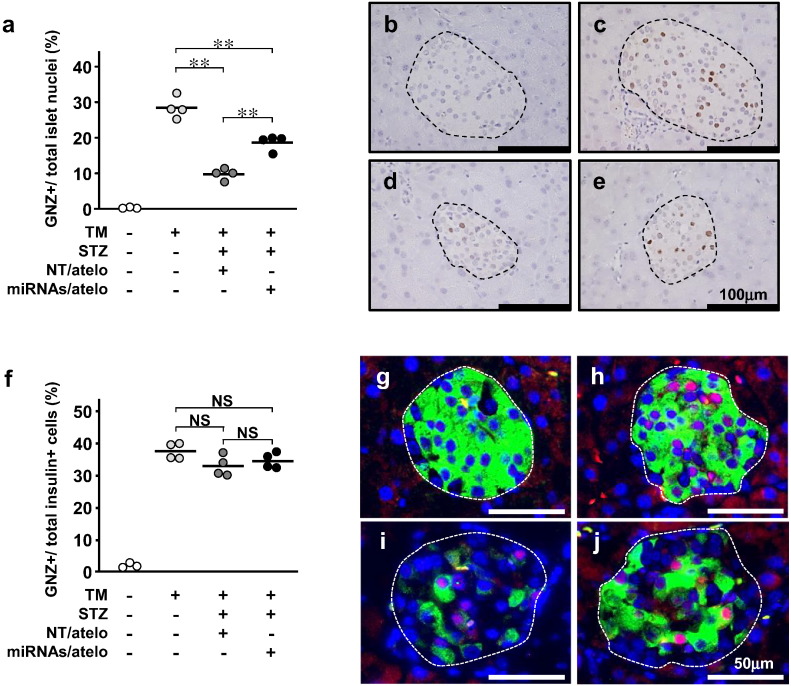
Intravenous administration of miR-106b and miR-222 stimulates β-cell proliferation. (a) Percentage of GNZ-positive islet nuclei. Each circle represents a single mouse. (b–e) Representative images of pancreatic islets stained for GNZ with anti-GFP antibody from TM-untreated DTg-mice (b), TM-treated DTg-mice (c), TM-treated STZ-NC-DTg-mice (d) and TM-treated STZ-miRNA-DTg-mice (e); islets are surrounded by a dashed line. (f) Ratios of GNZ/insulin-positive cells to total insulin-positive cells. Each circle represents a single mouse. (g–j) Representative images of pancreatic islets stained for GNZ (red), insulin (green), and DAPI (blue). GNZ and DAPI double-positive nuclei appear pink. TM-untreated DTg-mice (g), TM-treated DTg-mice (h), TM-treated STZ-NC-DTg-mice (i) and TM-treated STZ-miRNA-DTg-mice (j). * *p* < 0.05, ** *p* < 0.01, NS, not significant.

Finally, to further examine whether the origin of proliferating β-cells is β-cells per se or non-β progenitor cells, we performed the lineage-tracing procedure. In RIP-CreER™-mice, the insulin promoter drives expression of tamoxifen (TM)-dependent Cre recombinase (Dor et al., 2004). In Rosa26-GNZ-mice, Cre-mediated removal of the loxP-flanked STOP sequence results in expression of a GFP/LacZ fusion protein (GNZ). GNZ is expressed exclusively in the nuclei, because it has a nuclear localization signal (NLS) (Stoller et al., 2008). RIP-CreER™-mice and Rosa26-GNZ-mice were crossed to generate double transgenic mice (DTg-mice). When TM is injected into DTg-mice, GNZ is expressed in the nuclei of insulin-expressing cells. GNZ will also be expressed in the nuclei of progeny cells derived from GNZ expressing cells (Supplementary Fig. 4a). When 4-week-old DTg-mice were intraperitoneally injected with TM for 5 consecutive days to label β-cells, GNZ was selectively expressed in the nuclei of islet cells (Supplementary Fig. 4b). Starting at age 6 weeks, mice underwent STZ-treatment followed by miRNAs/atelo injection. Pancreatic islets were analyzed at age 9 weeks (Supplementary Fig. 4c). TM-treated DTg-mice had 28.0 ± 1.5% GNZ-positive cells in islets (Fig. 4a and c). TM-treated STZ-NT-DTg-mice had a significantly lower percentage of GNZ-positive islet cells (9.5 ± 0.9%) than TM-treated DTg-mice (Fig. 4a and d), indicating STZ-induced β-cell death. In contrast, TM-treated STZ-miRNA-DTg-mice had a significantly higher percentage of GNZ-positive islet cells than TM-treated STZ-NT-DTg-mice (Fig. 4a and e). In addition, the ratios of GNZ-positive cells among insulin-producing cells (β-cells) were similar among the three TM-treated groups (Fig. 4f–j), indicating a minimal contribution of new β-cells derived from non-β cells. Thus, proliferation of cells already producing insulin, i.e. β-cells per se, is the predominant mechanism of β-cell regeneration induced by miRNA mimic administrations.

## 4 Discussion

First, to elucidate the mechanism of BMT-induced β-cell regeneration, we comprehensively examined miRNA levels in serum exosomes from BMT-mice and found two increased miRNAs, miR-106b and miR-222, which reportedly suppress expressions of Cip/Kip family members. Administration of anti-miRNAs specific for these miRNAs suppressed β-cell regeneration and elevated blood glucose after BMT following STZ treatment, suggesting their involvement in BMT-induced β-cell regeneration. Intriguingly, intravenous administration of synthetic mimics of these miRNAs promoted β-cell proliferation after STZ treatment, thereby achieving recovery from insulin-deficient diabetes. In addition, the lineage tracing procedure revealed proliferation of β-cells per se after intravenous miRNA administration. Thus, the mechanism involving miRNAs apparently underlies post-injury β-cell regeneration induced by BMT and is a potential therapeutic target of regenerative medicine for diabetes.

In past years, a potential role of BM in diabetes therapy was discussed from a different viewpoint. A number of groups examined utilization of BM cells as a source of β-cells, focusing especially on whether BM cells directly differentiate into insulin-producing cells in response to BMT. Although those groups found that direct differentiation of BM cells into β-cells is highly unlikely, they fortuitously provided highly important experimental evidence indicating that BM cells are capable of facilitating endogenous regeneration of recipient β-cells, potentially by secreting trophic factors (Hasegawa et al., 2007, Hess et al., 2003). Despite intensive research, however, the potent secretory protein(s)/peptide(s) promoting β-cell regeneration after BMT has not yet been identified. Recently, exosomes, secreted by mesenchymal stem cells of BM cells, were reported to enhance regeneration of cells in several organs, including the heart, kidneys and liver (Barile et al., 2014, Zhou et al., 2013, Herrera et al., 2010), although the underlying molecular mechanism(s) has yet to be fully determined. In this study, we demonstrated that treatment with two anti-miRNAs inhibited BMT-induced β-cell regeneration, while intravenous administration of the corresponding miRNA mimics promoted post-injury β-cell proliferation through down-regulation of cell cycle inhibitory molecules. Lineage tracing experiments revealed that the cells which proliferated in response to systemic administration of these miRNA mimics had already produced insulin, i.e. they were β-cells. Thus, miR-106b and miR-222, which BM cells secrete higher amounts of in response to BMT, are involved in the mechanism underlying BMT-induced β-cell regeneration. After BMT, BM-derived cells were identified around regenerating islets (Hess et al., 2003, Hasegawa et al., 2007). Therefore, exosomal miR-106b-5p and miR-222-3p might be transferred from these BM-derived cells infiltrating the areas around the regenerating islets, in addition to via the circulation, although further examinations are required to clarify what type(s) of BM cells, such as hematopoietic stem cells, endothelial progenitor cells or mesenchymal stem cells, secrete exosomes, how BMT enhances exosome secretion and how the secreted exosomes are selectively delivered to β-cells.

Several reports have indicated links between β-cell mass and miRNAs which cell-autonomously function within β-cells. For instance, miR-338-3p is reportedly decreased in β cells of pregnant or obese rats (Jacovetti et al., 2012). It has also been reported that systemic knockout of miR-375 reduces β-cell mass in young (4-week-old) mice (Poy et al., 2009), while β-cell-specific knockout of miR-200 enhances β-cell apoptosis in obese diabetic mice (Belgardt et al., 2015). In addition to these cell-autonomous regulations, miRNAs are preferentially packaged in exosomes, which are secreted into the extracellular space and/or peripheral circulation and then taken up by neighboring or distant cells. miRNAs, delivered by exosomes, are thought to play an active role in inter-cellular and inter-tissue communication (Turchinovich et al., 2013, Hirsch et al., 2013). Therefore, based on previous reports (Hess et al., 2003, Nakayama et al., 2009), including ours (Hasegawa et al., 2007), indicating that BMT promotes β-cell regeneration, we screened exosomal miRNAs after BMT and newly identified a pair of miRNAs which are involved in BMT-induced β-cell proliferation. We further found that intravenous administration of these miRNA mimics resulted in efficient delivery to islet cells. Selective injury of β-cells induced by STZ treatment may lead to efficient and selective delivery of both exosomal miRNAs and miRNA mimics to pancreatic β-cells. As a result, Cip/Kip family members, which are known to function as cell cycle inhibitors, are downregulated in β-cells, thereby promoting β-cell proliferation in mice with insulin-deficient diabetes.

In this study, we demonstrated that β-cell proliferation can be stimulated by exogenous administration of miRNAs in vivo. β-cells have post-injury regenerative capacity, but the processes are slow and highly restricted (Bouwens and Rooman, 2005). Particularly, in human adult pancreatic β-cells, Cip/Kip family members, such as p27^kip1^ (Stein et al., 2014, Stein et al., 2013) and p57^kip2^ (Avrahami et al., 2014), reportedly play key roles in maintaining quiescence, leading to very limited proliferation in response to the numerous mitogens, growth factors, and nutrients that have been proposed to induce β-cell expansion (Kulkarni et al., 2012). Therefore, the identification of these miRNAs, which suppress Cip/Kip family members in β-cells, might overcome this important obstacle to therapeutic application of β-cell regeneration. It should be noted that the sequences of miR-106b-5p and miR-222-3p are identical in mice and humans. Therefore, the present results have major potential in the development of regenerative therapies for diabetes mellitus.

The following are the supplementary data related to this article.

Supplementary Table 1 miRNome analysis of serum exosomal miRNA expression in BMT mice versus control mice (without irradiation and infusion of bone marrow cells)
The fold change of microRNA expression in the BMT mice are indicated in columns labeled expression levels
A higher number indicates upregulation in the BMT mice and a lower number indicates downregulation. Supplementary Table 1

Supplementary Table 2 Fold change of the upregulated and downregulated miRNAs in the miRNome analysis.
The fold change of microRNA expression in the BMT mice are Indicated in columns labeled expression levels.
A higher number indicates upregulation in the BMT mice and a lower number indicates downregulation.
Listed are the miRNAs that expression changed > 3-fold in the miRNome analysis. Supplementary Table 2

Supplementary figures Image 2

## Conflict of Interest

There are no conflicts of interest to this study.

## Author Contributions

S.T., S.H., H.T., T.Y. performed the experiments. S.T., T.Y. and H.K. conceived the study. T.Y. and H.K. supervised the study. S.T., T.Y. and H.K. wrote the paper. K.T., J.G., Y.M., Y.S., S.K., Y.A., T.S., Y.C., K.K., K.U., S.S. and J.I. contributed to the interpretation of the data.

## Acknowledgments & Funding Sources

We thank T. Takasugi, K. Namiki, S. Fukasawa, M. Hoshi and J. Fushimi for technical assistance. This work was supported by Grants-in-aid for Scientific Research (to S.T, T.Y. and H.K.) from the Japan Society for the Promotion of Science (16K19528 to S.T., 26293215 to T.Y., 25253067 to H.K.) . This work was also supported by a Grant-in-Aid for Scientific Research on Innovative Areas (to H.K.) from the Ministry of Education, Culture, Sports, Science and Technology of Japan (22126006 to H.K.).
