# Supplementary material for: MicroRNAs 106b and 222 Improve Hyperglycemia in a Mouse Model of Insulin-Deficient Diabetes via Pancreatic β-Cell Proliferation
Source: eBioMedicine. 2016 Dec 7;15:163–72. doi: 10.1016/j.ebiom.2016.12.002 (PMC5233820; doi:10.1016/j.ebiom.2016.12.002)
Supplement: Supplementary file 3 — Supplementary figures [file mmc3.pdf]

Supplementary Figure 1.

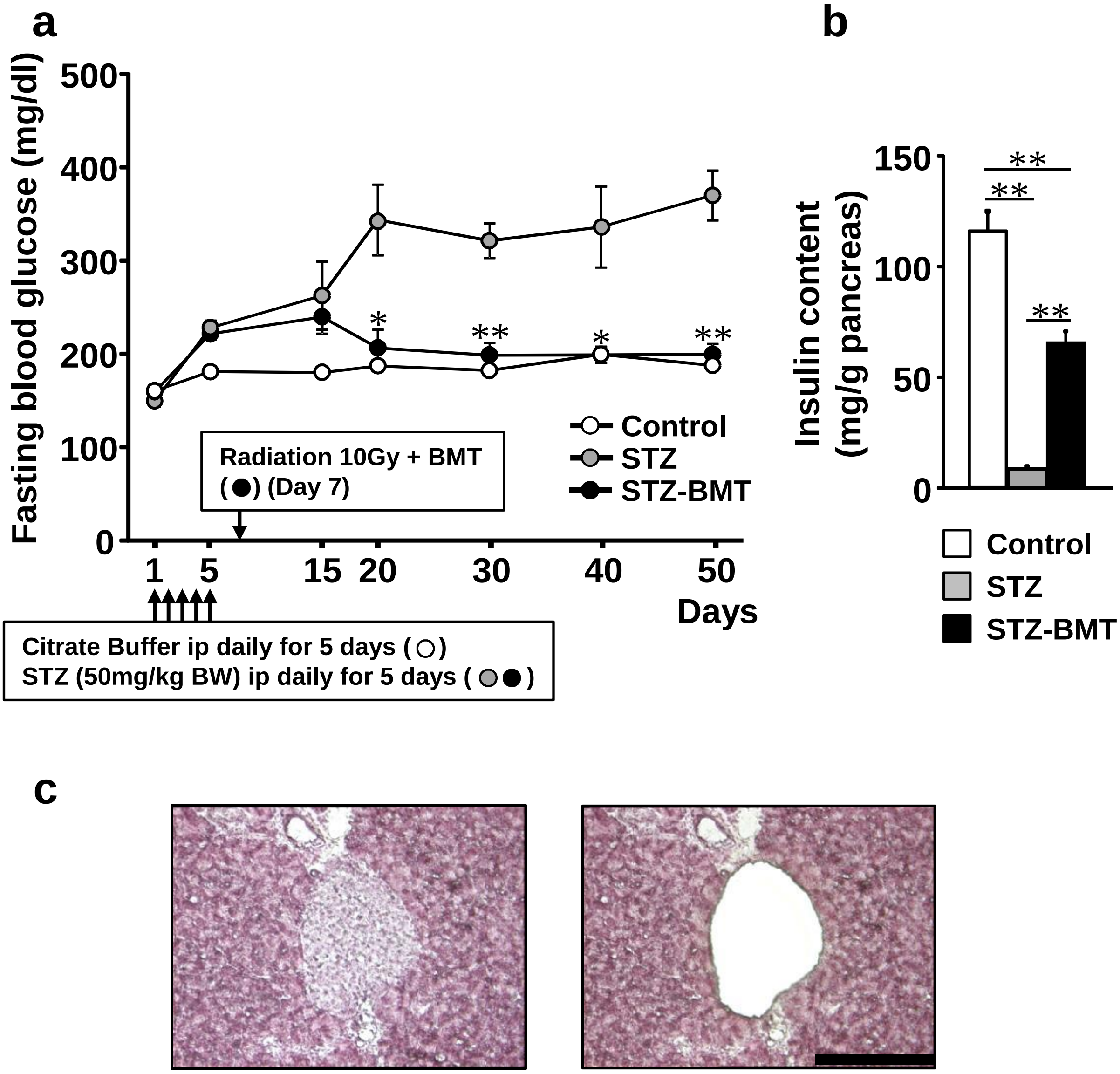

Supplementary Figure 1. BMT improves hyperglycemia in STZ-treated mice.

(**a**) Fasting blood glucose levels of STZ-treated mice with and without BMT. Control, citrate buffer-treated mice with neither STZ nor BMT. STZ, STZ-treated mice without BMT. STZ-BMT, STZ-treated mice receiving BMT (BM cells were infused after lethal irradiation). Control n=6, STZ n=5, STZ-BMT n=6. *p* value for STZ versus STZ-BMT. Data are presented as means ± SEM; and * $p < 0.05$, ** $p < 0.01$.

(**b**) Pancreatic insulin content on day 50 after the first STZ administration. Control n=6, STZ n=5, STZ-BMT n=6. Data are presented as means ± SEM; and ** $p < 0.01$.

(**c**) Hematoxylin staining of a pancreatic section before (left panel) and after (right panel) laser-microdissection of pancreatic islet. Scale bar, 200μm.

Supplementary Figure 2.

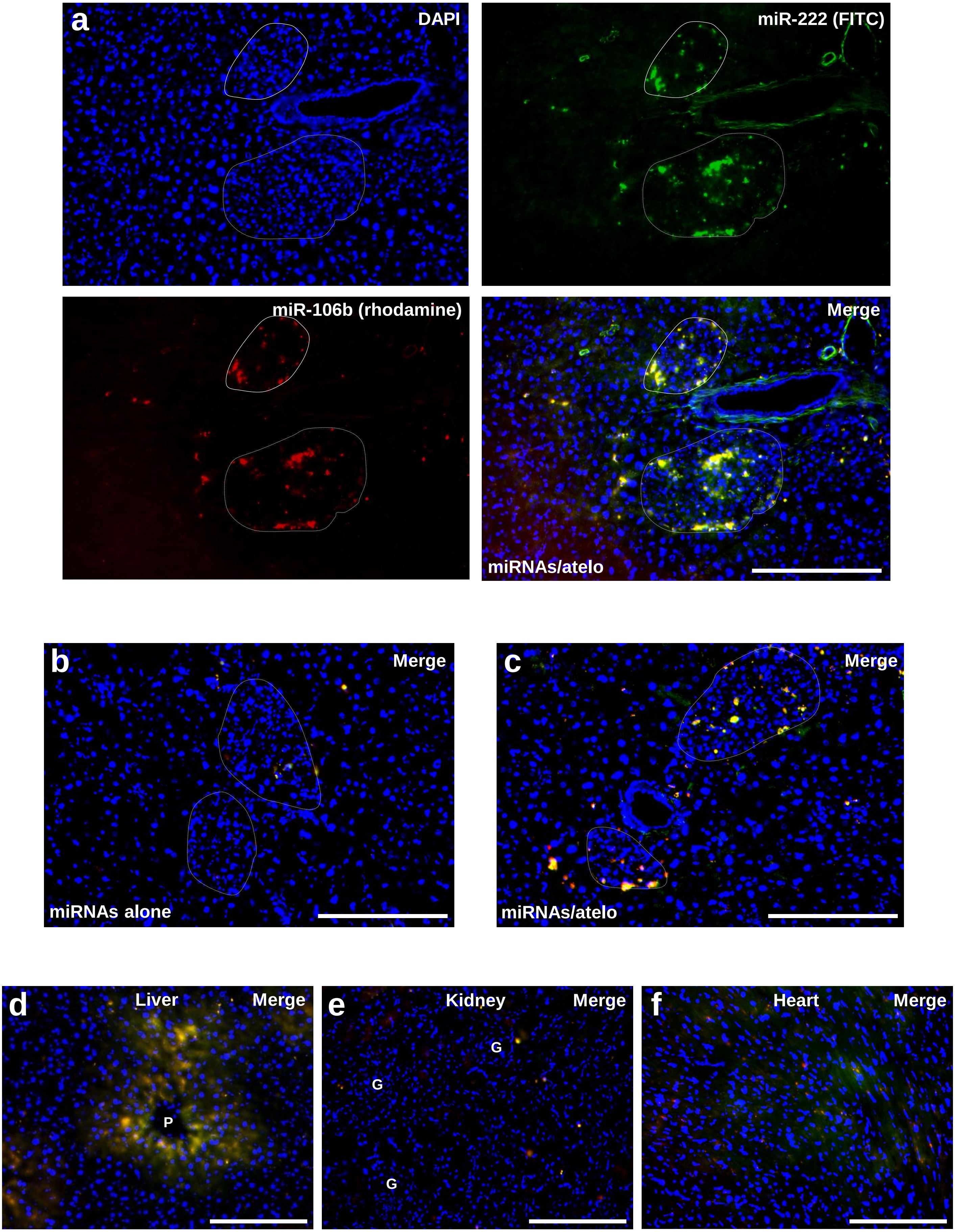

Supplementary Figure 2. miRNA mimics can be efficiently delivered to islet cells of STZ-treated mice by atelocollagen-mediated intravenous injection.

(**a, b**) Representative images of pancreatic cryostat sections at 24 hours after injection of fluorescein-labeled miRNA mimics. Mice received i.p. injections of STZ for 5 consecutive days starting on day 1. On day 5, rhodamine-labeled miR-106b (red) and FITC-labeled miR-222 (green), with or without being mixed with atelocollagen, were injected into the tail veins of STZ-treated mice. In merged images, the yellow color denotes the accumulation area of miRNA mimics. Strong fluorescence was observed in islets at 24 hours after injection of miRNA mimics with atelocollagen (miRNAs/atelo) (**a**), while fluorescence was scarcely observed in islets after injection of miRNA mimics alone (miRNAs alone) (**b**); islets are surrounded by a dashed line. Scale bar, 200μm.

(**c**) Representative merged images of cryostat sections at 48 hours after injection of miRNAs/atelo. Modest fluorescence was observed in islets at 48 hours after miRNAs/atelo injection. Islets are surrounded by a dashed line. Scale bar, 200μm.

(**d-f**) Representative merged images of cryostat sections of the liver (**d**), kidney (cortex) (**e**), and heart (left ventricular wall) (**f**) at 24 hours after injection of miRNAs/atelo. Diffuse fluorescence was observed around the portal vein (**d**), while fluorescence was scarcely observed in the kidney (**e**) and heart (**f**). P, portal vein; G, glomerulus. Scale bar, 200μm.

Supplementary Figure 3.

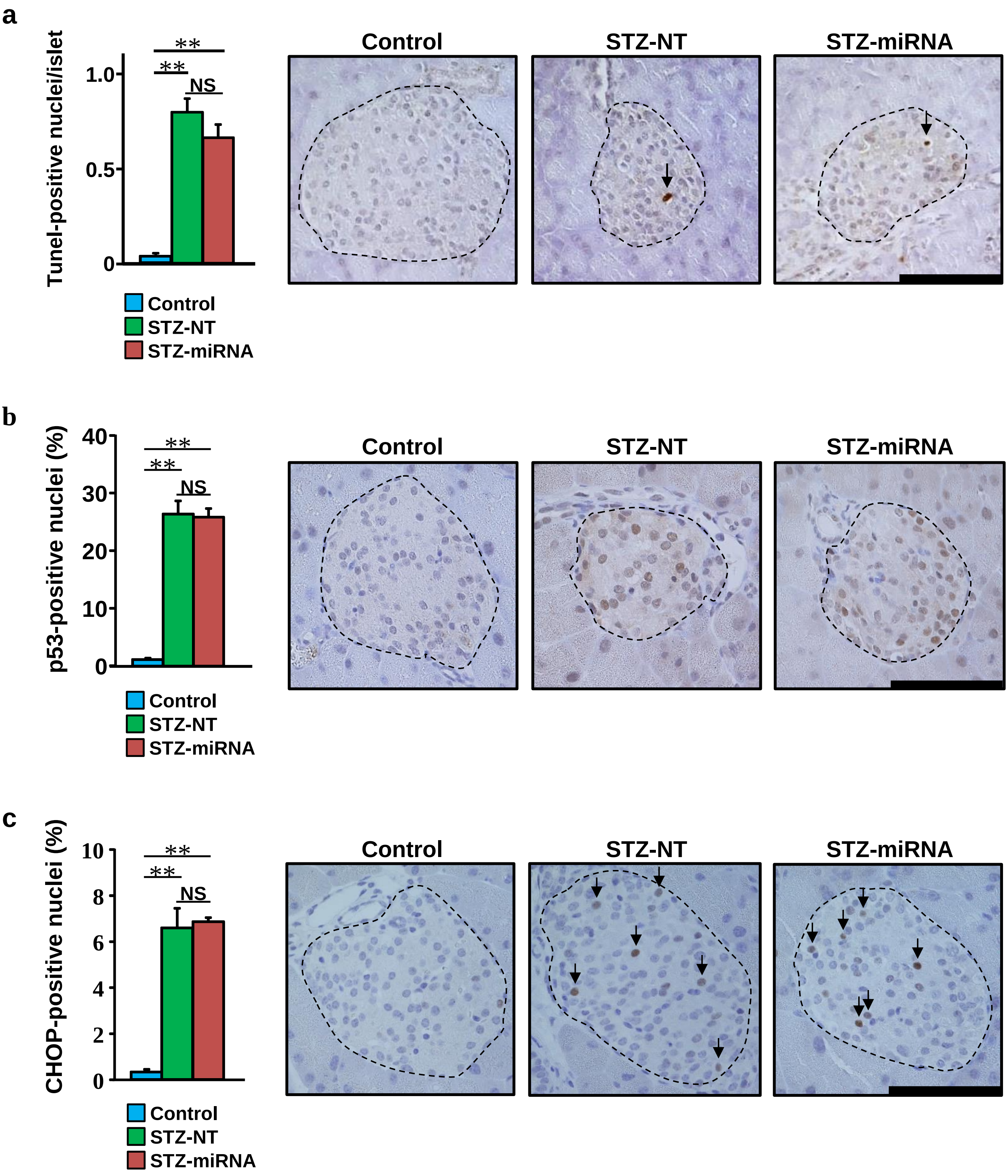

Supplementary Figure 3. Intravenous administration of miR-106b and miR-222 has little effect on apoptosis of β-cells after STZ treatment.

(**a-c**) Mice received i.p. injections of STZ for 5 consecutive days starting on day 1. Non-targeting control mixed with atelocollagen (STZ-NT) or miR-106b and miR-222 mimics mixed with atelocollagen (STZ-miRNA) were intravenously injected into STZ-mice on days 5 and 8. Mice were sacrificed for histological analysis on day 9. Scale bar, 100 μm.

(**a**) The apoptotic index (The mean number of TUNEL-positive nuclei per islet) (left) and representative images of pancreatic islets stained for TUNEL (right) on day 9; arrowhead indicates a TUNEL-positive nucleus. n=4 per group. Data are presented as means ± SEM; and ** $p < 0.01$, NS, not significant.

(**b**) Percentage of p53-positive islet cells (left) and representative images of pancreatic islets stained for p53 (right) on day 9; islets are surrounded by a dashed line. n=4 per group. Data are presented as means ± SEM; and ** $p < 0.01$, NS, not significant.

(**c**) Percentage of CHOP-positive islet cells (left) and representative images of pancreatic islets stained for CHOP (right) on day 9; arrowhead indicates a CHOP-positive nucleus. n=4 per group. Data are presented as means ± SEM; and ** $p < 0.01$, NS, not significant.

Supplementary Figure 4.

a

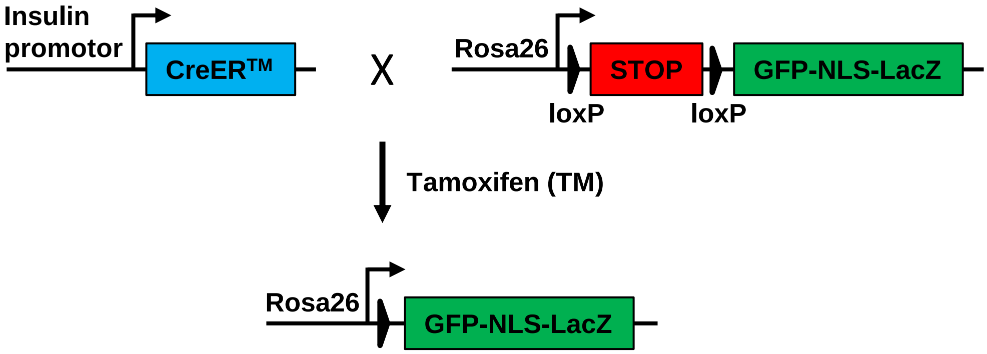

b

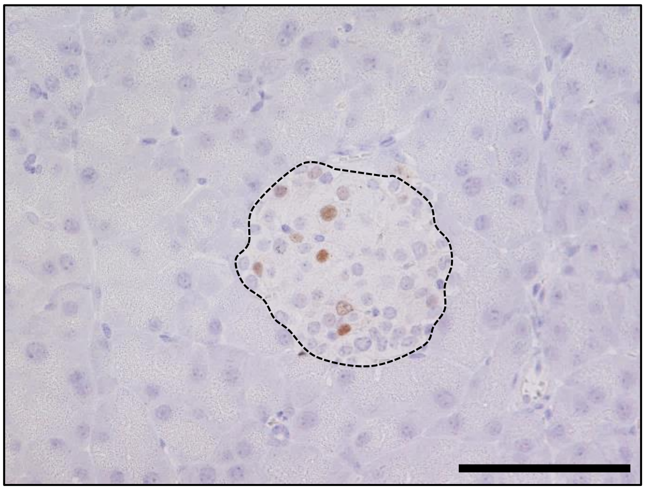

c

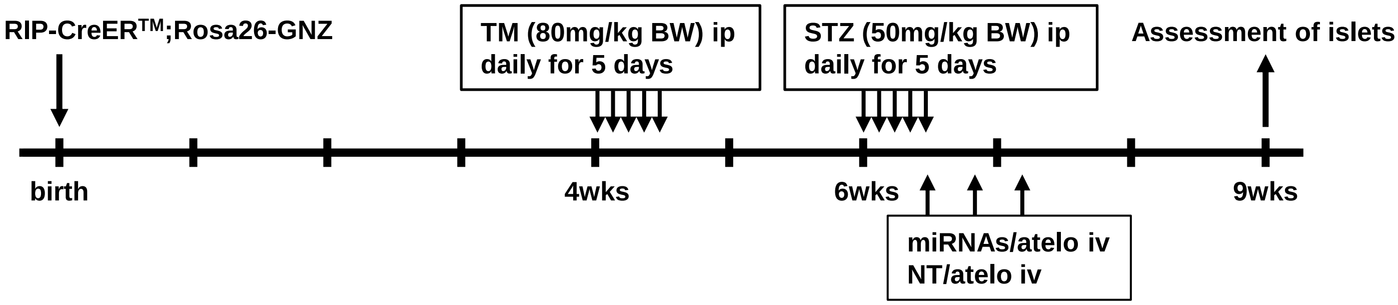

Supplementary Figure 4. Experimental design of β-cell lineage tracing in RIP-CreER^{TM}; Rosa26-GNZ mice.

(**a**) Strategy for the generation of a mouse line with TM-inducible β-cell-specific expression of GNZ protein.

(**b**) Representative image of pancreatic islet stained for GNZ with anti-GFP antibody. TM-treated male double transgenic mice (TM-treated DTg-mice) were sacrificed for histological analysis at 9 weeks of age. Scale bar, 100 μm.

(**c**) Schematic representation of the experimental design. At 4 weeks of age, male DTg-mice were injected with TM. After TM-treatment, the mice underwent STZ treatment followed by systemic injection of miRNA mimics as indicated.
